# Does temporal predictability of tasks influence task choice?

**DOI:** 10.1007/s00426-020-01297-1

**Published:** 2020-02-17

**Authors:** V. Jurczyk, V. Mittelstädt, K. Fröber

**Affiliations:** 1 Department of Psychology, University of Regensburg, Universitätsstr. 31, 93053 Regensburg, Germany; 2 Department of Psychology, University of Tübingen, Tübingen, Germany

## Abstract

Task performance improves when the required tasks are predicted by the preceding time intervals, suggesting that participants form time-based task expectancies. In the present study, we pursued the question whether temporal predictability of tasks can also influence task choice. For this purpose, we conducted three experiments using a hybrid task-switching paradigm (with two tasks) combining forced-choice and free-choice trials. Each trial was preceded by either a short (500 ms) or a long (1500 ms) foreperiod. In forced-choice trials, the instructed task was predicted by the length of the foreperiod (Exp. 1A and 1B: 100% foreperiod-task contingencies; Exp. 2: 80% foreperiod-task contingencies). In the remaining trials, participants were free to choose which task to perform. In all three experiments, we found that participants’ task choice was influenced by the foreperiod-task contingencies implemented in forced-choice trials. Specifically, participants were overall biased to choose tasks compatible with these contingencies; these compatible choice rates were larger for the short compared to the long foreperiod. Our findings suggest that learned time-based task expectancies influence subjects’ voluntary task choice and that an initially present task bias toward the “short” task is not always overcome at the long foreperiod. We discuss potential underlying mechanisms against the background of voluntary task switching and interval timing.

## Introduction

The timing of events is an important predictor for action planning and action selection (cf., Kolling & O'Reilly, 2018; Petter, Gershman, & Meck, 2018): Imagine meeting with a friend. While you are waiting at your arranged meeting place, time goes by. At first, you will probably be prepared for your friend to be on time; your planned course of action accordingly is to interact with that person. If you are waiting for some minutes, you will start to overthink that plan. May be, it would be wise to call or message your friend in case they forgot or had another meeting place in mind? Thus, depending on the time passing by, different events and also different actions will become more likely. As a consequence, your readiness for doing certain tasks should slowly shift from one action to the next as time goes by. There are many cases in which the relationship between passing time and expected events has high informative value for task selection. Previous research has shown that participants learn the temporal predictabilities of events as reflected in observable changes in performance. For example, task performance improves for trials in which the foreperiod is predictable of the upcoming task (i.e., so-called time-based task expectancies; Aufschnaiter, Kiesel, Dreisbach, Wenke, & Thomaschke, 2018). However, it is unclear whether this information is also actively used to guide task selection. In the present study, we, therefore, aim to investigate whether and how temporal predictability can also influence task-choice behavior.

### General and specific temporal predictability effects

Within our introduction, we will first review the previous studies demonstrating influences of temporal predictability on performance before turning to a discussion of contextual influences on task-choice behavior. Finally, we will elaborate on whether and how time-based task expectancies may influence task-choice behavior. In general, timing studies have primarily focused on investigating the expectancy of certain time durations (general time expectancy; Los & Heslenfeld, 2005; Niemi & Näätänen, 1981), conscious time estimation (interval timing; Balcı & Simen, 2016), and the neural underpinnings of time perception (see, e.g., Merchant & Lafuente, 2014; Wearden, 2016). One basic finding is that subjects can learn to incorporate the expected duration of a foreperiod in their behavior: If the foreperiod duration is predictable or precued, performance (e.g., reaction time, RT) is improved in predicted compared to unpredicted or unpredictable intervals (Coull & Nobre, 1998; Coull, Frith, Büchel, & Nobre, 2000; Los, 2013; Miniussi, Wilding, Coull, & Nobre, 1999). Interestingly, if the interval duration is fixed within a block, performance profits from short intervals compared to long foreperiods (the fixed foreperiod effect, Niemi & Näätänen, 1981); however, with variable foreperiods, performance improves with prolonged foreperiod durations (the variable foreperiod effect; e.g., Los & Heslenfeld, 2005; Los & Van den Heuvel, 2001; Steinborn, Rolke, Bratzke, & Ulrich, 2008). A large portion of this latter effect can be ascribed to an asymmetric sequential effect, in that short-foreperiod trials suffer from a preceding long-foreperiod trial, whereas long-foreperiod trials are answered fast no matter the preceding foreperiod length (Los, Kruijne, & Meeter, 2014; Steinborn et al., 2008; Vallesi & Shallice, 2007). On a theoretical level, the effects can be most parsimoniously accounted for by a trace conditioning account (Los et al., 2014; Los & Heslenfeld, 2005): The beginning of the foreperiod (often indicated by a warning stimulus) marks the beginning of a temporally structured cascade where unspecific readiness to respond is shaped by a conditioning process. The conditioned strength of each time point is reinforced whenever the imperative stimulus appears at that time point. It remains unchanged whenever the foreperiod ends before passing that time point and is weakened when that time point passes without an imperative stimulus appearing. Other accounts favor a more strategic point of view (Niemi & Näätänen, 1981), assuming that participants actively prepare according to the estimated probability of stimulus occurrence, or a mixture of both intentional preparation as well as unintentional conditioning (Langner, Steinborn, Eickhoff, & Huestegge, 2018; Vallesi & Shallice, 2007).

More relevant for the purpose of the present study are findings of temporal expectancies for specific events (Wagener & Hoffmann, 2010). A growing body of literature shows that time–event correlations can be exploited for improving behavior: if an event appears more likely after a certain foreperiod (than after another foreperiod, or than another event), this foreperiod–event correlation will influence behavior: expected time-event associations will lead to better performance (faster RTs, smaller error rates) than unexpected ones (Thomaschke, Wagener, Kiesel, & Hoffmann, 2011). Thus, in contrast to the variable foreperiod effect eluded to above, the time–event correlation paradigm aims at temporally manipulating readiness to respond in a specific way, rather than shaping unspecific readiness. It has been shown for events such as specific stimulus–response associations (Schröter, Birngruber, Bratzke, Miller, & Ulrich, 2015), stimulus–effector associations (Thomaschke & Dreisbach, 2013), and even affective qualities (Bogon, Thomaschke, & Dreisbach, 2017). These time-based event expectancy effects were also found robustly across different frequency distributions of events and foreperiods and across different foreperiod durations (Thomaschke et al., 2011).

On a theoretical level, the time–event correlation effect is presumably due to learning associations between intervals and the respective stimulus–response events (Thomaschke & Dreisbach, 2015). Participants then use these learned expectations to prepare for the required task or event, depending on the passing time. Several studies suggest that it is primarily action preparation rather than perceptual preparation that profits from those time–event correlations (Thomaschke & Dreisbach, 2013, 2015; Volberg & Thomaschke, 2017). For example, when a certain effector (e.g., the right digit finger) is more often required after a certain time interval (e.g., after a short than after a long foreperiod), responses using this effector will be faster even when controlling for stimulus–time associations (Thomaschke & Dreisbach, 2013). While these studies suggest that action preparation is an important contributing factor to time-based expectancy effects, they do not preclude the other forms of preparation to play a role, too. For example, (task) performance is also improved if the upcoming stimulus location can be predicted based on the time interval (compared to an unpredicted stimulus location; Pfeuffer, Aufschnaiter, Thomaschke, & Kiesel, 2020; Wagener & Hoffmann, 2010).

### Evidence for time-based (task) expectancies

Importantly though, specific time expectancy goes beyond simple time–effector correlations: Providing evidence for this, Wendt and Kiesel (2011) have found that if high- or low-response-conflict likelihood is associated with a short or long foreperiod, respectively, attentional adjustments will be stronger if the current foreperiod predicts large conflict likelihood and vice versa. Another example is provided by recent studies demonstrating the formation of time-based task expectancies (Aufschnaiter, Kiesel, Dreisbach et al., 2018; Aufschnaiter, Kiesel, & Thomaschke, 2018). These studies make use of a version of the task-switching paradigm in which a cue indicates which task to perform on a given trial (e.g., if a target number is displayed in blue, participants should perform a parity classification; if presented in red, they should perform a magnitude classification). Critically, the task can either switch or repeat from one trial to the next. The classic finding is that performance (RTs and/or error rates) is worse in switch trials compared to repetition trials (for reviews, see Monsell, 2003; Kiesel et al., 2010; Vandierendonck, Liefooghe, & Verbruggen, 2010). These so-called switch costs indicate that additional time is needed to reconfigure a new task set and/or overcome the previously required one. Consequently, switch trials especially profit from longer (temporally nonpredictive) preparation intervals (e.g., Monsell & Mizon, 2006). Here, the elapsed time neither serves as a cue for unspecific readiness (as in the variable FP paradigm) nor as a task-specific trigger (as in the time-event correlation paradigm). Instead, passing time just means more time for passive decay of the old (interfering) task-set and if a specific task cue is given more time to prepare for the upcoming task.

To investigate the influence of predictive preparation intervals on task performance, Aufschnaiter, Kiesel, Dreisbach et al. (2018; see also Aufschnaiter, Kiesel, & Thomaschke, 2018 ) combined this task-switching procedure with the variable foreperiod paradigm by implementing contingencies between foreperiods and tasks: Participants were presented with digits that were either to be classified according to their parity or according to their magnitude (smaller or larger than five) depending on the color of the appearing digit. Importantly, one task was more often preceded by one foreperiod (e.g., parity task after a short foreperiod) and the other task was more often preceded by another foreperiod (e.g., magnitude task after long), while the overall frequency of short and long foreperiods was kept equal. Over three experiments that differed in terms of the degree of predictability (90%, 80%, and 70%), subjects were faster (and in Experiment 1 also less error-prone) in trials with frequent foreperiod–task associations. This effect of temporal predictability did not depend on awareness.

### Contextual influences on task-choice behavior

Critically, in these studies, the specific task to be performed in a given trial was always specified (e.g., a color indicated the appropriate task in a trial so-called forced-choice trials). However, temporal predictability in our environment may also be used when we voluntarily decide which course of action to pursue. Following up on the above-mentioned example of meeting with an unpunctual friend, the passing time provides information about which task is most appropriate (e.g., making a phone call as time passes). In general, people are usually free which task they want to perform, and thus, they need to flexibly schedule whether they perform the same task again or whether they want to switch to another task (e.g., Fröber & Dreisbach, 2017). Recent research suggests that people are able to adapt their task-choice behavior to changing multitasking environments, such as changes in rewards for task completion (Fröber & Dreisbach, 2016) or to predictable changes in task availabilities (Mittelstädt, Miller, & Kiesel, 2018). However, as far as we are aware, there are no previous studies investigating whether and how time-based task expectancy influences voluntary task choice.

This question is not trivial: to further our theoretical understanding of time-based task expectancies, it is important to understand not only how they influence task performance, but also task-selection processes. Task performance and task selection seem to involve partially diverting cognitive control mechanisms (Arrington & Yates, 2009; Chen & Hsieh, 2013; Orr & Weissman, 2011). Consequently, even though studies have found factors influencing task-choice behavior such as preparation time (Arrington & Logan, 2004), stimulus repetition (Mayr & Bell, 2006), or task difficulty (Yeung, 2010), these effects are not directly deducible from task-performance effects: For example, when confronted with the voluntary choice between two tasks that vary in task difficulty, participants perform better in the relatively easier task, while task choice is biased toward the more difficult task.

In voluntary task choice, action selection seems to precede stimulus selection (Herbort & Rosenbaum, 2014). Thus, variables that primarily affect action selection (e.g., differential response interference; Jurczyk, Fröber, & Dreisbach, 2018; Yeung, 2010) should influence free-choice behavior to a greater degree than variables affecting stimulus selection (e.g., by specifying the stimulus, but not the response hand, Herbort & Rosenbaum, 2014). Time-based expectancy effects largely reflect the expectancy of certain actions (Volberg & Thomaschke, 2017), suggesting that a modulating influence on task choice is possible.

### The present experiments

The present study aims to investigate people’s voluntary task-choice behavior in a temporally structured task environment. For this purpose, we will use the hybrid task-switching paradigm (i.e., a combination of free-choice and forced-choice trials) introduced by Fröber and Dreisbach (2016, 2017): Here, in each block, there is a combination of both free- and forced-choice trials. Using univalent stimuli, trials where just one stimulus of one task appears constitute forced-choice trials, while two appearing stimuli of both tasks indicate free choice. If free-choice trials are paired with a sufficient ratio of forced-choice trials, a reasonable amount of voluntary switching can be obtained (e.g., voluntary switch rates of over 20% with 50% forced-choice trials; Fröber, Raith, & Dreisbach, 2018) even without telling participants explicitly to do so (as opposed to instructing participants to do both tasks equally often but in random order, see Arrington & Logan, 2004, 2005; Arrington, Reiman, & Weaver, 2014). Thus, in contrast to the standard voluntary task-switching paradigm, task-choice behavior can be investigated without additional instructions that restrict participants’ choice behavior.

Of importance, we varied contingencies between time intervals and tasks for forced-choice trials (see Aufschnaiter, Kiesel, Dreisbach et al., 2018). The main question which we are pursuing is whether temporal contingencies implemented in forced-choice trials influence task choice in free-choice trials. Based on findings of temporal predictability effects on task performance and findings that people are able to adapt their task-choice behavior to different task environments, task-choice behavior may be biased toward tasks compatible with the foreperiod–task associations formed in forced-choice trials. On the other hand, as mentioned earlier, it is not clear whether task-choice behavior is biased at all in this temporally structured environment.

It is in particular interesting to explore how participants will adapt their task choice behavior to the different foreperiod lengths. Depending on whether the size of the effect differs between the short and the long foreperiod, we can make tentative inferences about the underlying mechanisms. If the compatible choice rate is higher for the longer foreperiod, participants presumably rather built time-based task expectancies for the long foreperiod-task association. By design, as long as the short foreperiod has not passed, the probabilities for the foreperiod to continue or of a stimulus to be shown are equal (for a similar argument along the lines of hazard function in the variable foreperiod effect, see, e.g., Nobre, Correa, & Coull, 2007). The long foreperiod, however, will always be followed by a stimulus display, most likely of one specific task. Also, even in voluntary task switching, preparation processes profit from longer pretarget intervals (Arrington & Logan, 2004). This might extend to processes related to temporal predictability.

On the other hand, compatible choice rate might also be stronger or of the same size for the short foreperiod: In the variable foreperiod paradigm, sequential and modality-based modulations are restricted to the short foreperiod (Steinborn, Rolke, Bratzke, & Ulrich, 2009, 2010). For task performance, time-based task-expectancy effects for the short foreperiod were found, and sometimes even resulted in an RT benefit for this short foreperiod. Aufschnaiter et al. (2018) speculate that this may be due to higher phasic alertness after the short foreperiod (cf., Meiran, Chorev, & Sapir, 2000; Niemi & Näätänen, 1981) or less precise time-keeping ability the more time passes (the scalar property of timing, for a review, see Hass & Durstewitz, 2014). However, one could also argue that if participants start out the trial preparing for one task, they may not always be able to switch preparation to the other task (see also “General Discussion”). Finally, the temporal predictabilities established in the forced-choice trials might affect voluntary choice behavior on the level of general task preferences rather than in a fine-grained manner depending on the foreperiod: as participants were completely free in their task-choice behavior which makes the emergence of such task biases more likely (Kessler, Shencar, & Meiran, 2009), we also checked whether systematic overall task biases emerged.

In our analyses, we also considered whether participants switched or repeated tasks, because these task transitions have considerable effects on both task performance and task choice. Specifically, robust switch costs are also present in voluntary task-switching settings and participants usually show a strong bias to repeat tasks (Arrington & Logan, 2004, 2005; Fröber & Dreisbach, 2017; Kessler et al., 2009). Time-based task-expectancy effects on performance are sometimes stronger for task switches, but no clear picture has emerged yet. The present findings might help to further our understanding of the interaction between task transition and temporal predictability.

To foreshadow, even though participants’ choice behavior was overall biased to select the compatible task in Experiment 1, the specific length (short vs. long) of the foreperiod additionally modulated task choice. In Experiment 2 and 3, we demonstrate the robustness of these (partially surprising) timing-induced choice biases across modified paradigms.

## Experiment 1A

In the first experiment, the contingencies between foreperiods and tasks in the forced-choice trials were kept at 100%; thus, on forced-choice trials one task was always preceded by a short foreperiod (i.e., 500 ms), while the other task was always preceded by a long foreperiod (i.e., 1500 ms, Aufschnaiter, Kiesel, Dreisbach et al., 2018). A first learning block consisted only of those forced-choice trials. In the subsequent hybrid test phase, we used a 50:50 free-choice to forced-choice ratio—free-choice trials were randomly preceded by short and long foreperiods. Participants were instructed to voluntarily select one of the two possible tasks in each free-choice trial (i.e., without any randomness instruction as in typical voluntary task-switching studies; for a review, see Arrington et al., 2014). The percentage of choices compatible with the temporal predictabilities of the forced-choice trials (hereafter compatible choice rate) was the main dependent variable. The hybrid test phase was followed by a 100% forced-choice test block where foreperiods were no longer predictive of the upcoming task. Thus, this final phase allowed us to assess whether typical time-based task-expectancy effects on forced-choice task performance (i.e., better performance for predicted compared to unpredicted foreperiod-task associations; Aufschnaiter, Kiesel, Dreisbach et al., 2018; Aufschnaiter, Kiesel, & Thomaschke, 2018) can also be observed in this hybrid task-switching environment.

Although our main goal was to investigate whether temporal predictability influences task-choice behavior, we also investigated whether time-based task expectancies on forced-choice trials will influence task performance on free-choice trials in a similar manner (i.e., faster RTs for expected interval-task combinations than for unexpected interval-task combinations). Even though this is not a necessary preliminary for effects on task choice, the analysis provides an important extension of previous time-based task-expectancy effects from forced-choice to free-choice trials. If so, this would demonstrate that time-based expectancy effects on task performance presumably build on similar mechanisms both for forced-choice and free-choice trials.

## Methods

### Participants

Thirty-two students of the University of Regensburg took part in this study (26 female; 26 right-handed; age range = 18–26; *M* = 20.2; SD = 2.0) for course credit or money (6 €). We chose this number of participants on the basis of the main effect of temporal predictability on task performance reported in a previous study (Aufschnaiter, Kiesel, Dreisbach et al., 2018). A sample size of 26 has proven sufficient to detect the effect with a level of statistical power of 80% and a significance level of 5% using the bias- and uncertainty-corrected sample size planning tool available at www.DesigningExperiments.com (see also Anderson, Kelley, & Maxwell, 2017). The software allows to conduct power analysis based on a previous empirical effect while correcting for publication bias and uncertainty (we assumed an assurance level of 50%). For reasons of counterbalancing, we rounded to 32 participants. All participants signed informed consent prior to the experiment and were naïve with respect to the purpose of the experiment. Participants were treated in accordance with the ethical standards of the American Psychological Association.

### Apparatus

The experiment was run using E-Prime 2.0 (Psychology Software Tools, Sharpsburg, PA) on a 19-inch TFT display (display resolution at 1280 × 1024, refresh rate 60 Hz). Responses were collected with a QWERTZ-keyboard, using “y” and “x” as left and right response keys for one task (left hand), and “n” and “m” as keys for the other task (right hand). Responses were to be given with the digit and middle finger of the respective hand. Participants were seated at approximately 60 cm from the screen (unconstrained), at which distance 1 cm on the screen corresponds approximately to 1° of visual angle.

### Stimuli and procedure

Stimuli consisted of numbers (125, 132, 139, 146, 160, 167, 174, 181) or letters (B, D, F, H, S, U, W, Y), which had to be categorized as smaller or larger than 153 (number task) or nearer to A or nearer to Z in the alphabet (letter task) by pressing a left or right response key, respectively. We used this seemingly arbitrary number task (opposed to the typically used smaller/larger than five tasks) to match tasks in terms of difficulty.^1^ Stimuli of one task appeared always above a central fixation cross and stimuli of the other task below (1.3 cm). Mapping of number or letter task to position on the screen was fixed but counterbalanced across participants, while responses to the upper task were always given with the left hand. All stimuli were displayed in black ink (Calibri font, size 28, bold) on a silver (RGB: 192, 192, 192) background. A central fixation cross was displayed in Calibri font, size 24, bold, 0.4 cm. On forced-choice trials, only one stimulus appeared on screen; on free-choice trials, two stimuli were shown simultaneously and participants were free to choose which task to perform (voluntary task switching).

Participants practiced both tasks separately in two short practice blocks (16 trials each so that each stimulus randomly appeared two times, order of tasks counterbalanced across subjects, already paired with the respective foreperiods, see below). These were followed by one practice block of forced-choice switch trials (16 trials). A subsequent longer learning block of 128 trials also consisted of just forced-choice trials and was meant to establish foreperiod-task contingencies: one task was always paired with a 500 ms foreperiod (short fixation duration) and the other with a 1500 ms foreperiod (long fixation duration) prior to stimulus onset. The foreperiod-task contingencies were fixed during the experiment for each participant, but were counterbalanced across participants. A following hybrid test phase consisted of five blocks of 128 trials each. 50% of the trials were forced-choice trials, 50% free-choice trials. All stimuli appeared equally often, but without direct stimulus repetitions, and the order of stimuli was pseudorandomized, so that all combinations of trial type (forced, free) × foreperiod/task (short, long; numbers, letters) appeared about equally often and equally distributed. In the free-choice trials, each foreperiod appeared with equal probability, while also controlling for equally distributed foreperiod-foreperiod transitions.

**Fig. 1:**
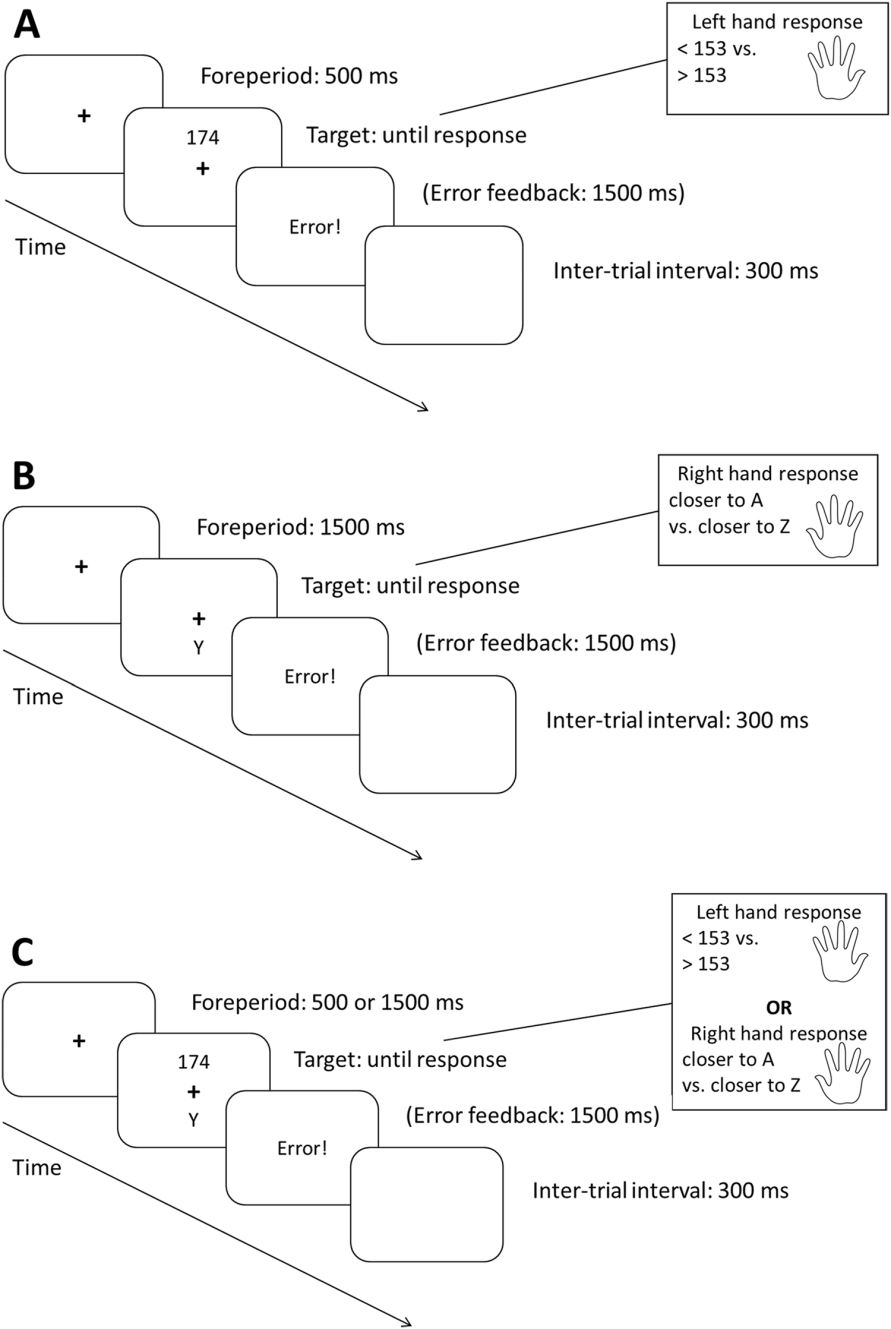
Illustration of a single trial. A: Example of a forced-choice trial with a short foreperiod, followed by one of the tasks. B: Example of a forced-choice trial with a long foreperiod, followed by the other task. C: Example of a free-choice trial with variable foreperiod, short or long, and voluntary choice between both tasks

The trial procedure for both forced-choice and free-choice trials is depicted in Fig. 1. Each trial began with the presentation of the fixation cross for either 500 ms (short) or 1500 ms (long). Then, the stimulus or, for free-choice trials, the stimuli were presented until a response was made. A feedback display was only displayed if an error was made. It lasted for 1500 ms and the word “Fehler!” (German for “Error”) was displayed. The intertrial interval was a blank screen and lasted for 300 ms.

In a last 100% forced-choice test phase (another block of 128 trials), the contingencies between tasks and foreperiods were abolished, meaning that each task was preceded by the short foreperiod in 50% of trials, and by the long foreperiod in the remaining 50% of the trials.

### Design

Task-choice behavior on the free-choice trials in the test phase was of main interest, especially whether participants preferentially chose the task compatible with the foreperiod-task contingencies implemented on forced-choice trials. Compatible choice rate was defined as the percentage of trials in which participants chose the task compatible with the current foreperiod (as established in forced-choice trials). This compatible choice rate was assessed via one-sample *t* tests against compatibility choice rates predicted by chance (i.e., 50%) as well as in a more fine-grained 2 (foreperiod: short, long) × 2 transition: repetition, switch) repeated-measures analysis of variance (ANOVA). We also analyzed via one-sample *t* tests (against a 50% chance level) whether general choice biases emerged: Task biases toward the task associated with the short or long foreperiod and the repetition bias were considered.

Furthermore, we analyzed RTs and ERRs on free-choice trials in 2 (foreperiod) × 2 (compatibility: compatible vs. incompatible task choice) × 2 (transition) repeated-measures ANOVA.

In the last test block (forced-choice trials only), RTs and ERRs were analyzed as a function of foreperiod (short, long), previous compatibility (compatible, incompatible) of the current foreperiod-task association, and transition (repetition, switch).

## Results

### Data preprocessing

Raw data of this and the following experiments can be found under https://epub.uni-regensburg.de/41403/. We excluded the first trial of each block (0.8%), as it does not entail task transition. Task-choice analyses used all remaining trials including errors to cover all attempts of voluntary switching (cf. Arrington & Logan, 2004). Errors in free-choice trials were assigned to task by selected hand as it can be assumed that participants rather choose the wrong finger than choose the wrong hand (Scheffers & Coles, 2000). In the RT analyses, we additionally excluded trials with excessively slow or fast reaction times (over 3 *SD*s from the subject’s mean in a condition; 1.8% of all trials), error trials (4.6%), as well as post-error trials (4.3%). We further excluded the data set from two participants as they were considered outliers (as identified by boxplots) in mean ERRs.^2^

### Free-choice trials: task choice

We first examined whether general task biases occurred. Overall, participants showed a bias toward the task associated with the short foreperiod (*M* = 56.6, SD = 27.7), *t*(29) = 11.19, *p* < 0.001, *d* = 0.24, and also toward the number task (*M* = 52.2, SD = 28.4), *t*(29) = 10.07, *p* < 0.001, *d* = 0.08. They repeated tasks more often than they switched (24.3% switches, SD = 12.2), *t*(29) = − 11.56, *p* < 0.001, *d* = − 2.11. A paired *t* test between switch rates of the foreperiod conditions indicated that this repetition bias was stronger for the short foreperiod (short: 22.6% switches vs. long: 26.1% switches), *t*(29) = -3.46, *p* = 0.002, *d* = -0.26.

**Fig. 2:**
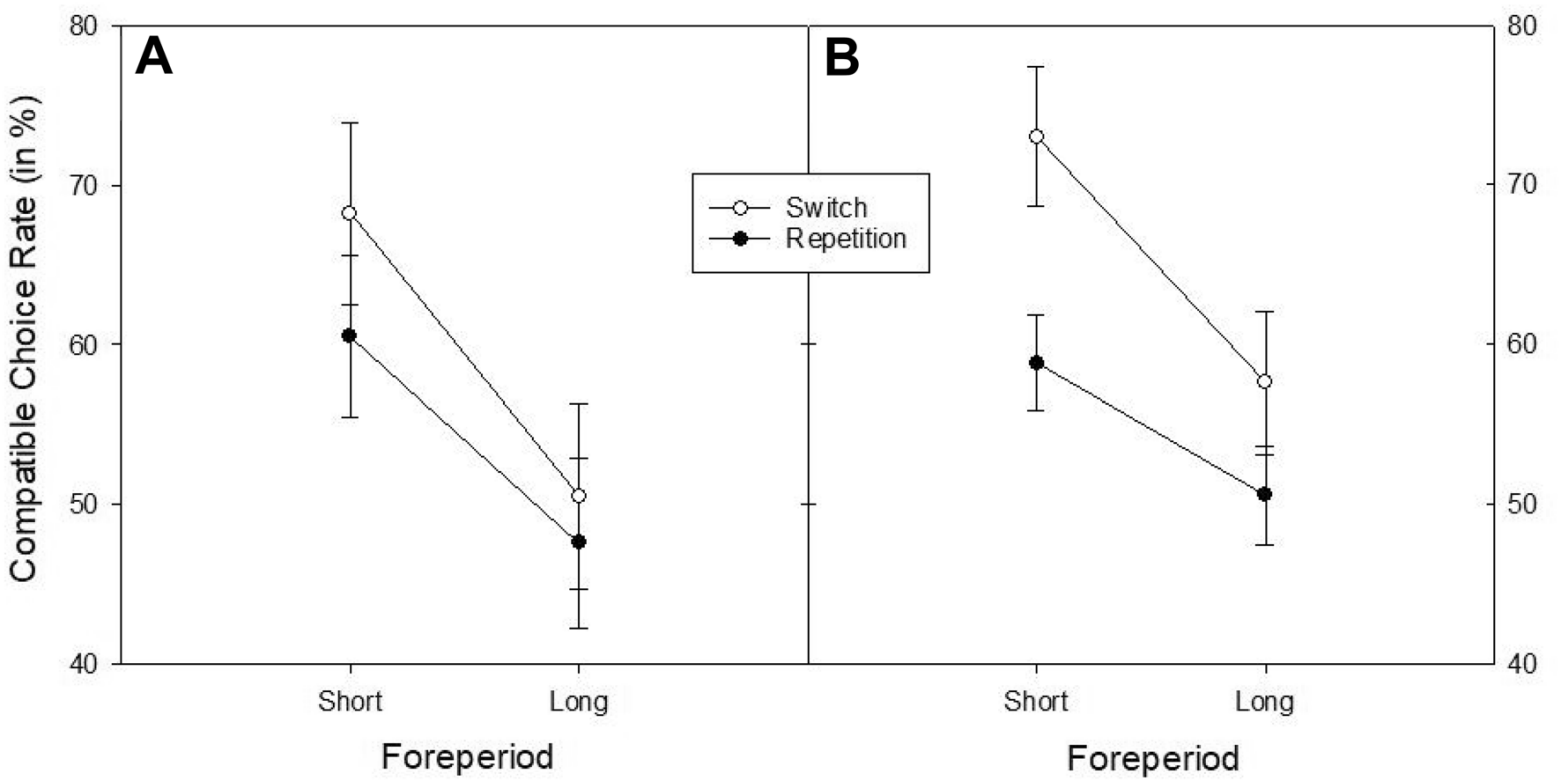
Compatible choice rate (in %) in Experiment 1A (panel A) and 1B (panel B) as a function of foreperiod and transition. Error bars represent ± 1 SEM

Most importantly with respect to our research question, a *t* test showed that participants chose the compatible task overall more often than predicted by chance (*M* = 55.2, SD = 7.6), *t*(29) = 3.75, *p* < 0.001, *d* = 0.69.^3^ This effect was present for the short foreperiod (*M* = 61.8, SD = 7.6), *t*(29) = 12.13, p < 0.001, *d* = 0.42, but not the long foreperiod (*M* = 48.7, SD = 7.61), *t*(29) = 9.02, p < 0.001, *d* = − 0.05, where overall the “short” task was still chosen more often than the “long” task. In a 2 × 2 ANOVA on compatible choice rate with the factors foreperiod and transition (see Fig. 2a), only the main effect of transition was significant, *F*(1, 29) = 12.83, *p* = 0.001, $\eta_{p}^{2}$ = 0.31. The compatible choice rate was higher on voluntary switch trials as compared to voluntary repetition trials (59.4% vs. 54.0%). No other effect reached significance (both *F*s < 2.2; both *p*s > 0.14).

### Free-choice trials: RTs and ERRs

RTs and ERRs for all conditions are shown in Table 1. The 2 (foreperiod) × 2 (compatibility) × 2 (transition) repeated-measures ANOVA on RTs revealed significant switch costs, *F*(1, 21) = 36.59, *p* < 0.001, $\eta_{p}^{2}$ = 0.64, further qualified by an interaction of transition and compatibility, *F*(1, 21) = 4.35, *p* = 0.049, $\eta_{p}^{2}$ = 0.17. The compatibility effect only showed up on repetition trials, with slower repetitions after an incompatible foreperiod. No other effect reached significance (all *F*s < 2.00, all *p*s > 0.180).

**Table 1:** Mean RTs (in ms) and ERRs (in %) in free-choice trials of Experiment 1A as a function of foreperiod (short vs. long), compatibility (compatible vs. incompatible), and transition (repetition vs. switch)

	Short foreperiod				Long foreperiod			
	Compatible		Incompatible		Compatible		Incompatible	
	Repetition	Switch	Repetition	Switch	Repetition	Switch	Repetition	Switch
RT (SD)	674 (83.4)	827 (140)	715 (89.9)	841 (150)	677 (112)	818 (203)	704 (105)	789 (119)
ERR (SD)	4.24 (3.93)	1.88 (4.17)	3.43 (4.16)	5.50 (8.23)	2.26 (1.75)	1.36 (2.44)	4.34 (4.62)	4.32 (5.09)

The same analysis on ERRs yielded only a significant effect of compatibility, *F*(1, 21) = 5.47, *p* = 0.029, $\eta_{p}^{2}$ = 0.21. Furthermore, a marginally significant interaction of compatibility and transition, *F*(1, 21) = 3.36, *p* = 0.081, $\eta_{p}^{2}$ = 0.14, was found. Compatibility effects were only found for switch trials. All other effects were nonsignificant (all *F*s < 2.00, all *p*s > 0.170).

### Forced-choice trials: final test block

In the last forced-choice only block, foreperiod was no longer predictive of the upcoming task. A 2 (foreperiod) × 2 (previous compatibility) × 2 (transition) repeated-measures ANOVA on RTs revealed both a main effect of previous compatibility (676 ms vs. 693 ms for compatible and incompatible trials, respectively), *F*(1, 29) = 5.07, *p* = 0.032, $\eta_{p}^{2}$ = 0.15, and of transition (633 ms vs. 736 ms), *F*(1, 29) = 42.68, *p* < 0.001, $\eta_{p}^{2}$ = 0.60. Foreperiod and transition interacted significantly, *F*(1, 29) = 4.71, *p* = 0.038, $\eta_{p}^{2}$ = 0.14, reflecting larger switch costs at short (117 ms) compared to the long foreperiod (89 ms). Compatibility effects showed a tendency to be larger on repetition trials as compared to switch trials (28 ms vs. 8 ms, respectively), but the interaction between compatibility and transition was only marginally significant, *F*(1, 29) = 3.00, *p* = 0.094, $\eta_{p}^{2}$ = 0.09.

The analogous ERR ANOVA only showed a significant interaction of compatibility by transition, *F*(1, 29) = 8.22, *p* = 0.008, $\eta_{p}^{2}$ = 0.22, driven by a large compatibility effect of 2.2% on repetition, and a descriptively reversed effect of -0.9% on switch trials.

## Discussion

On free-choice trials, participants showed a substantial bias to choose the task compatible with the current foreperiod (i.e., the task associated with the current foreperiod on forced-choice trials). This effect was especially pronounced on switch trials. The compatibility effect found in task choice was mirrored in free-choice performance, with smaller RTs (on repetitions) and lower ERRs for compatible foreperiod-task pairings. In a final forced-choice only test block (without foreperiod-task correlations), we tested whether time-based task-expectancy effects on forced-choice task performance could be replicated (Aufschnaiter, Kiesel, Dreisbach et al., 2018; Aufschnaiter, Kiesel, & Thomaschke, 2018) in our task environment: Indeed, task-to-foreperiod pairings that matched previously learnt ones resulted in faster RTs and better ERRs.

Interestingly, participants showed a tendency to preferably do the task associated with the short foreperiod—a tendency that was reduced, but still descriptively present, after the long foreperiod. Even though this may very well lie in the mechanics of how time-based task-expectancy works (see “General Discussion”), a single new finding has to be treated with caution. Therefore, before drawing strong conclusions about the generality of this “short” task bias,^4^ Experiment 1B sought out whether this “short” task bias was also present with an increased ratio of forced-choice trials.

## Experiment 1B

In Experiment 1A, we observed that participants were biased to select the task compatible with the presented foreperiod. Interestingly, a general bias toward the task associated with the short foreperiod (a “short” task bias) was also observed. However, this latter effect was primarily present in some participants with very strong task biases. The main aim of Experiment 1B was to investigate whether a “short” task bias will also be present in a setting with a higher ratio of forced-choice trials (75:25). Fröber and Dreisbach (2017) have shown that a larger variability in terms of task choices and thus, less extreme task biases—can be achieved by this manipulation: when forced to switch tasks frequently, a context of increased cognitive flexibility is established that transfers to the free-choice trials. Furthermore, we expected to again observe an overall preference for the compatible task. Given that a higher ratio of forced-choice trials means a higher ratio of trials where the foreperiod is predictive of the upcoming task, it is very well possible that participants’ tendency to select the compatible task will be larger in Experiment 1B as compared to Experiment 1A.

We further included a post-questionnaire to ask participants if they could consciously recall the foreperiod-task association and, if so, whether they deliberately used it for their task-choice behavior. If participants become aware of the foreperiod-task contingencies, this may open the way to an explicit, conscious strategy of using these associations for free choice (e.g., Bijleveld, Custers, & Aarts, 2012). Previous studies have shown that specific temporal predictability does not critically depend on awareness (Aufschnaiter, Kiesel, Dreisbach et al., 2018; Aufschnaiter, Kiesel, & Thomaschke, 2018). Even though these findings are not suggestive of it, there is some indication that this might be different under voluntary task-switching conditions, given that it involves more intentional, top-down control (Arrington & Logan, 2005).

## Methods

### Participants

Thirty-two participants of the Albert Ludwigs University of Freiburg took part in this study (23 female, 31 right-handed, age range = 20–30; *M* = 23.8, SD = 2.6) for course credit or money (6 €). All participants signed informed consent prior to the experiment and were naïve with respect to the purpose of the experiment.

### Apparatus, stimuli, procedure, and design

Everything was exactly the same as in Experiment 1A except for the following changes. In the test phase of combined free-choice and forced-choice trials, the ratio of forced-choice to free-choice trials was now set to 75:25. Additionally, participants filled in a short questionnaire after the experiment asking them whether they were aware of the foreperiod-task contingencies and, if so, whether they used this knowledge for their task-choice behavior.^5^

## Results

### Data preprocessing

Exclusion criteria were the same as in Experiment 1A and resulted in the removal of 0.8% of the data (for RT analysis: 11.2%).

### Free-choice trials: task choice

We first checked for general biases in task choice behavior. Participants chose the task associated with the short foreperiod more often than predicted by chance (*M* = 62.4, SD = 21.1), *t*(26) = 3.32, *p* = 0.002, *d* = 0.59. There was no significant bias toward either the number or letter task (bias toward number task: *M* = 51.5, *SD* = 24.5), *t*(31) = 0.36, *p* = 0.725, *d* = 0.06. Participants repeated tasks more often than they switched (switch rate: 29.7%, SD = 11.3), *t*(31) = − 10.18, *p* < 0.001, *d* = − 1.80; this repetition bias did not differ significantly between the short and long foreperiod (short: 28.7%, long: 30.7%), *t*(31) = − 1.54, *p* = 0.133, *d* = − 0.17.

Most important to our hypothesis, a *t* test again revealed a significant overall task choice bias toward the compatible task (*M* = 55.8, SD = 7.8), *t*(31) = 4.19, *p* < 0.001, *d* = 0.74. This compatible choice bias was significantly present after the short foreperiod (*M* = 68.1, SD = 20.5), *t*(31) = 5.00, *p* < 0.001, *d* = 0.88, and descriptively reversed after the long foreperiod (*M* = 48.5, *SD* = 24.3), *t*(31) = − 1.52, *p* = 0.138, *d* = − 0.27. The ANOVA on the compatible choice rate including foreperiod and transition as within-subjects factors revealed both a significant main effect of foreperiod, *F*(1, 31) = 13.15, *p* = 0.001, ${\upeta }_{{\text{p}}}^{{2}}$ = 0.30, and of transition, *F*(1, 31) = 22.62, *p* < 0.001, ${\upeta }_{{\text{p}}}^{{2}}$ = 0.43, as well as a marginally significant interaction between the two, *F*(1, 31) = 3.30, *p* = 0.079, ${\upeta }_{{\text{p}}}^{{2}}$ = 0.10 (see Fig. 2b). Again, compatible choice rate was larger on voluntary switch trials as compared to voluntary repetition trials (64.2% vs. 53.4%).

We again investigated whether the results on task choice differed when participants with an extreme task bias (participants with a bias toward one of the two tasks of > 95%^6^; 5 participants) were excluded. Importantly, while the “short” task bias was then no longer significant (55.5%), *t*(26) = 1.94, *p* = 0.063, *d* = 0.37, the compatible choice bias remained significant (56.9%), *t*(26) = 4.52, *p* < 0.001, *d* = 0.87.

In further analyses, we explored whether the differences in experimental manipulation between Experiment 1A and 1B significantly impacted free choice. However, neither in terms of overall compatible choice rate nor “short” task bias (neither on their own nor in a Duration × Transition ANOVA including the between-subjects factor Experiment) did the two experiments differ significantly (all *p*s > 0.330).

### Free-choice trials: RTs and ERRs

RTs and ERRs for all conditions are shown in Table 2. The ANOVA on RTs revealed the significant main effects compatibility, *F*(1, 22) = 9.55, *p* = 0.005, ${\upeta }_{{\text{p}}}^{{2}}$ = 0.30, and transition, *F*(1, 22) = 25.98, *p* < 0.001, ${\upeta }_{{\text{p}}}^{{2}}$ = 0.54, and a marginally significant effect of foreperiod, *F*(1, 22) = 3.93, *p* < 0.060, ${\upeta }_{{\text{p}}}^{{2}}$ = 0.15. Subjects were faster after a long foreperiod (806 ms vs. 833 ms), showed a compatibility effect (801 ms vs. 838 ms) and switch costs (746 ms vs. 894 ms). None of the interactions was significant (all *F*s < 2.70, all *p*s > 0.110).

**Table 2:** Mean RTs (in ms) and ERRs (in %) in free-choice trials of Experiment 1B as a function of foreperiod, compatibility, and transition

	Short foreperiod				Long foreperiod			
	Compatible		Incompatible		Compatible		Incompatible	
	Repetition	Switch	Repetition	Switch	Repetition	Switch	Repetition	Switch
RT (SD)	674 (83.4)	827 (140)	715 (89.9)	841 (150)	677 (112)	818 (203)	704 (105)	789 (119)
ERR (SD)	4.24 (3.93)	1.88 (4.17)	3.43 (4.16)	5.50 (8.23)	2.26 (1.75)	1.36 (2.44)	4.34 (4.62)	4.32 (5.09)

The same analysis on ERRs yielded a significant effect of foreperiod, *F*(1, 23) = 12.08, *p* = 0.002, ${\upeta }_{{\text{p}}}^{{2}}$ = 0.34. Participants made less errors after a long foreperiod (2.6% vs. 5.8%). Similar to Experiment 1A, a significant interaction of compatibility and transition arose, *F*(1, 23) = 4.37, *p* = 0.048, ${\upeta }_{{\text{p}}}^{{2}}$ = 0.16. A typical compatibility effect was found only for repetition trials, whereas a reversed effect was found for switch trials. All other effects were not significant (all *F*s < 1.90, all *p*s > 0.180).

### Forced-choice trials: final test block

In the last forced-choice only block, foreperiod-task contingencies were no longer valid. In the RT analysis, main effects of previous compatibility, *F*(1, 25) = 20.64, *p* < 0.001, $\eta_{p}^{2}$ = 0.45, transition, *F*(1, 25) = 35.19, *p* < 0.001, ${\upeta }_{{\text{p}}}^{{2}}$ = 0.59, as well as an interaction of foreperiod and transition, *F*(1, 25) = 13.72, *p* = 0.001, ${\upeta }_{{\text{p}}}^{{2}}$ = 0.35, were significant. Compatibility in the preceding test phase resulted in an advantage of 39 ms; switch costs were smaller after a long foreperiod, though this was mostly driven by slower RTs on long repetition trials. None of the other effects was significant (all *F*s < 0.40, all *p*s > 0.540).

ERRs showed only a significant main effect of transition (repetitions: 2.7% vs. switches: 5.4%), *F*(1, 31) = 16.49, *p* < 0.001, ${\upeta }_{{\text{p}}}^{{2}}$ = 0.35, with no other significant effects (all *F*s < 1.40, all *p*s > 0.250).

## Discussion

As in Experiment 1A, there was an overall significant compatible choice bias in Experiment 1B that tended to be stronger on switch compared to repetition trials. Thus, participants adapted their task-choice behavior to the current foreperiod and the task associated with it on forced-choice trials. Not only task choice, but also task performance on free-choice trials was influenced by the foreperiod-task correlations (in RTs, and repetition ERRs). In the last forced-choice only test block (i.e., without foreperiod–task correlations), previous compatibility still affected RTs.

Experiment 1B employed a higher ratio of forced-choice trials than Experiment 1A. Previous research (Fröber et al., 2018; Fröber & Dreisbach, 2017) has indicated that this increases the variability of task-choice behavior as it creates a context of increased cognitive flexibility. The same was true for the current experiments, as the overall voluntary switch rate increased from 24.3% in Experiment 1A to 29.7% in Experiment 1B. Yet in Experiment 1B, extreme task biases occurred with similar frequency as in Experiment 1A and were always directed toward the short foreperiod. Thus, this “short” task bias seems to reflect a systematic effect of temporal predictability on general task preference rather than being caused by a high number of free-choice trials. Additional analyses were run excluding participants with extreme task biases. These analyses rendered the “short” task bias insignificant, while the compatible choice bias was still present—suggesting that the former is largely driven by a few participants, whereas the latter is not.

## Experiment 2

The previous two experiments have established that temporal predictabilities indeed influence voluntary task-choice behavior: specifically, participants showed an overall bias to select the compatible task, but this compatible choice rate was modulated by a bias to select the task associated with the short foreperiod. An important limitation of the previous experiment is that 100% contingencies were used. This is rather atypical, particularly within time–event correlation paradigms (Aufschnaiter, Kiesel, Dreisbach et al., 2018; Thomaschke et al., 2011; Wagener & Hoffmann, 2010), and it could have influenced time-based task-expectancy processing in a different way than is normally seen in these paradigms. Also, in our everyday environment, contingencies are hardly ever perfect, but rather probabilistic. Thus, the purpose of Experiment 2 was to check whether the current findings would replicate in a task environment with more common contingencies (i.e., 80%). This also has the advantage that the difference between compatible and incompatible trials in forced-choice trials can be analyzed already within the test block.

## Method

### Participants

Another 32 participants of the University of Regensburg took part in this experiment for course credit or money (6 €). Of these, 30 were female and 28 were right-handed. They were between 19 and 34 years old (*M* = 22.7, SD = 3.4). All participants signed informed consent prior to the experiment, were naïve with respect to the purpose of the experiment, and did not participate in Experiment 1.

### Apparatus, stimuli, and procedure

Except for the following changes, everything was exactly the same as in Experiment 1A and 1B. On forced-choice trials, foreperiod–task contingencies were now fixed to 80%. The learning block was accordingly prolonged to 160 trials. The following test phase consistent of 5 blocks of 120 trials each; two third of the trials were forced-choice trials (again with 80% foreperiod–task contingency) and one third were free-choice trials. Since the compatibility effect on forced-choice trials could now already be calculated in the test block, the last forced-choice only test block was dropped. As in Experiment 1B after completing the experiment, participants filled in a short post-questionnaire regarding their awareness of the foreperiod-task contingencies.^7^

### Design

The design was analogous to Experiment 1A and 1B except that forced-choice trials in the hybrid test phase could already be analyzed in a 2 (foreperiod) × 2 (compatibility) × 2 (transition) repeated-measures ANOVA.

## Results

### Data preprocessing

Exclusion criteria were the same as in Experiment 1A and 1B and resulted in the removal of 0.8% of the data (for RT analysis: 10.9%).

### Free-choice trials: task choice

We again first checked for the emergence of general task biases. An overall bias toward the task associated with the short foreperiod was present (*M* = 62.0, *SD* = 20.9), *t*(31) = 3.25, *p* = 0.003, *d* = 0.58, while no bias toward either number or letter task emerged (bias toward number task: *M* = 54.2, SD = 23.9), *t*(31) = 0.99, *p* = 0.330, *d* = 0.18. A strong repetition bias was found (switch rate: *M* = 25.6, SD = 9.14), *t*(31) = -25.62, *p* < 0.001, *d* = − 2.67, which was again larger after a short foreperiod (short: 22.6% switches, long: 28.7% switches), *t*(31) = − 6.13, *p* < 0.001, *d* = − 0.60.

**Fig. 3:**
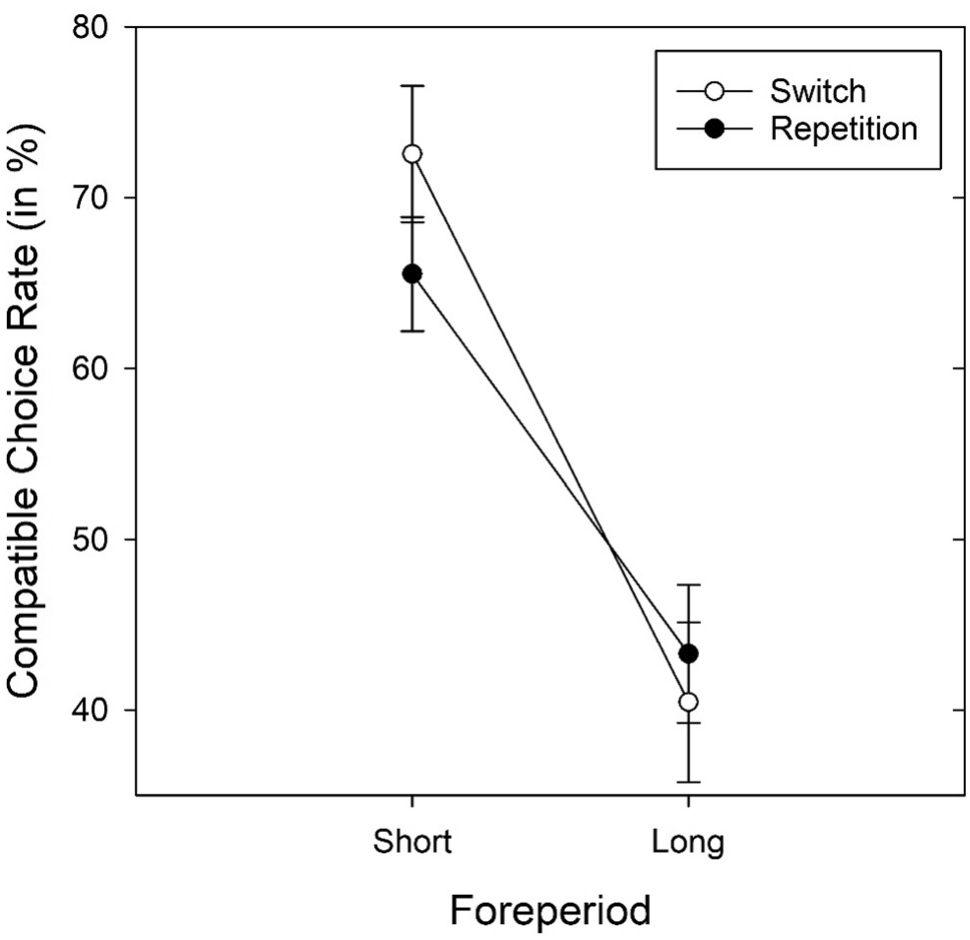
Compatible choice rate (in %) in Experiment 2 as a function of foreperiod and transition. Error bars represent ± 1 SEM

Crucially, an overall compatible choice bias was again significant (*M* = 54.4, SD = 3.99), *t*(31) = 6.30, *p* < 0.001, *d* = 1.11. It was stronger for the short foreperiod (*M* = 66.4, SD = 19.1), *t*(31) = 4.86, *p* < 0.001, *d* = 0.86, with a tendency toward the incompatible (“short”) task after the long foreperiod (*M* = 42.4, SD = 23.3), *t*(31) = − 1.85, *p* = 0.074, *d* = − 0.33. The ANOVA with the factors foreperiod and transitions revealed a main effect of foreperiod, *F*(1, 31) = 12.92, *p* < 0.001, ${\upeta }_{{\text{p}}}^{{2}}$ = 0.29, and an interaction of foreperiod and transition, *F*(1, 31) = 6.81, *p* = 0.014, ${\upeta }_{{\text{p}}}^{{2}}$ = 0.18 (see Fig. 3). Compatible choice rate was only significant for the short foreperiod. This effect was even stronger for voluntary switches. Five participants displayed very extreme task biases (> 95% bias toward one of the two tasks). Irrespective of whether it was a number or letter task, all these participants displayed a bias toward the “short” task. In an analysis excluding these participants, the “short” task bias was no longer significant (*M* = 55.4%), *t*(26) = 1.85, *p* = 0.076, *d* = 0.36, while there was still a significant bias to select the compatible task (*M* = 55.3%), *t*(26) = 7.37, *p* < 0.001, *d* = 1.42.

### Free-choice trials: RTs and ERRs

Mean RTs and ERRs can be found in Table 3. The RT ANOVA yielded only a significant main effect of transition, *F*(1, 23) = 34.47, *p* < 0.001, $\eta_{p}^{2}$ = 0.60, and a marginally significant main effect of compatibility, *F*(1, 23) = 4.07, *p* = 0.055, $\eta_{p}^{2}$ = 0.15. Participants showed switch costs of 128 ms and a trend compatibility effect of 24 ms. No other effect reached significance (all *F*s < 1.90, all *p*s > 0.190).

**Table 3:** Mean RTs (in ms) and ERRs (in %) in free-choice trials of Experiment 2 as a function of foreperiod, compatibility, and transition

	Short foreperiod				Long foreperiod			
	Compatible		Incompatible		Compatible		Incompatible	
	Repetition	Switch	Repetition	Switch	Repetition	Switch	Repetition	Switch
RT (SD)	674 (120)	830 (226)	725 (169)	822 (234)	691 (121)	800 (209)	699 (135)	846 (240)
ERR (SD)	3.07 (2.74)	3.78 (7.26)	1.94 (2.45)	6.68 (16.3)	4.24 (4.21)	2.80 (5.05)	2.91 (3.43)	9.91 (20.3)

The analogous ERR analysis revealed a marginally significant main effect of transition, *F*(1, 25) = 4.12, *p* = 0.053, ${\upeta }_{{\text{p}}}^{{2}}$ = 0.14, which was further qualified by an interaction with compatibility, *F*(1, 25) = 6.41, *p* = 0.018, ${\upeta }_{{\text{p}}}^{{2}}$ = 0.20. A substantial compatibility effect was only found on switch trials. All other effects were nonsignificant (all *F*s < 2.40, all *p*s > 0.130).

### Forced-choice trials

Divergent from the previous experiments, incompatible trials were already included in the test phase. Thus, performance on the forced-choice trials in the test phase were analyzed. We conducted Foreperiod × Compatibility × Transition ANOVAs on RTs and ERRs. For RTs, this analysis yielded a main effect of compatibility, *F*(1, 31) = 9.90, *p* = 0.004, $\eta_{p}^{2}$ = 0.24, as compatible trials were on average responded to 14 ms faster than incompatible trials, and a main effect of transition, *F*(1, 31) = 113.10, *p* < 0.001, ${\upeta }_{{\text{p}}}^{{2}}$ = 0.79, reflecting switch costs of 124 ms. Furthermore, the interaction of foreperiod and transition was significant, *F*(1, 31) = 30.70, *p* < 0.001, ${\upeta }_{{\text{p}}}^{{2}}$ = 0.50. Smaller switch costs arose after a long foreperiod, both owing to slower repetition RTs as well as faster switch RTs. No other effect was significant (all *F*s < 1.10, all *p*s > 0.330).

The analogous ERR analysis revealed only a main effect of transition, *F*(1, 31) = 39.13, *p* < 0.001, ${\upeta }_{{\text{p}}}^{{2}}$ = 0.56, with participants making errors in 5.89% of all switch trials, but only in 3.29% of all repetition trials. All other effects were nonsignificant (all *F*s < 0.80, all *p*s > 0.400).

## Discussion

The results of this experiment replicate the findings obtained in the previous two experiments and thus generalize them to a task environment with 80% foreperiod–task contingencies. In particular, a significant compatible choice bias was found which was larger for switch trials. Performance on free-choice trials was influenced by compatibility (marginally so in RTs, and only on switches for ERRs). Divergent from the previous two experiments, foreperiod–task contingencies were kept at 80% (and not 100%), thus allowing to compare compatible and incompatible forced-choice trials already in the hybrid task-switching phase. This comparison yielded significant RT compatibility effects, extending typical time-based task-expectancy effects on forced-choice trials to the free-choice context (Aufschnaiter, Kiesel, Dreisbach et al., 2018; Aufschnaiter, Kiesel, & Thomaschke, 2018).

Thus, Experiment 2 corroborates the finding of the previous experiments: A robust compatible choice bias was found, but was limited to (or stronger for) the short foreperiod. The compatible choice rate is larger for task switches. And overall, participants display a bias toward the task associated with the short foreperiod. This bias seems to be largely driven by a few participants with very strong task preferences.

## General discussion

In three experiments, we investigated whether task-choice behavior is biased by temporal cues in a temporally structured voluntary task environment. Specifically, in a task-switching setting forced-choice and free-choice trials were randomly intermixed (hybrid design) and the task in forced-choice trials was predicted by the foreperiod (Exp. 1A and 1B: 100% predictability; Exp. 2: 80% predictability). Our main interest was whether the randomly interspersed short and long foreperiods in free-choice trials biased task choice. Across all experiments, we found that compatible foreperiod-task combinations were chosen more often than incompatible ones. In the first two experiments, this effect was stronger on task-switch trials (in the last experiment, only for the short foreperiod, this dissociation was found). Furthermore, over all experiments, a compatible choice bias was only significant for the short foreperiod. Finally, temporal predictability also affected general task preferences: overall, participants were biased toward the task which was associated with the short foreperiod on forced-choice trials.

### Task-choice behavior and temporal preparation: a preparation-switch account

The present experiments provide the first evidence that time-based task-expectancy effects can not only be found for task performance, but also for task choice. As reviewed in the introduction, temporal predictability may influence task performance in forced-choice settings due to time-dependent changes in task preparation. In a similar vein, task-choice behavior might also be influenced by these changes in task preparation: Specifically, we suggest that task selection in our experiments utilized bottom-up biases as introduced by time-based task expectancy. Throughout the time course of the foreperiod, the preparatory activation level for either task changed as a function of temporal predictability which further translated to task selection (for a similar argument, see Arrington, 2008).

On a more functional level, this suggests that temporal predictability does not only influence the course of (active) task preparation, but also of task-selection processes. In studies on voluntary task switching, these two processes have been found to be distinguishable (Arrington et al., 2014), but in many cases, task preparation informs task selection (e.g., Arrington & Logan, 2004; Mittelstädt et al., 2018). On the one hand, while task preparation is mainly reflected in performance indices like the switch costs (in RTs and ERRs), task-selection processes are marked by task choice indices like the voluntary switch rate and task bias—and correlations between these markers have been found to be rather small (Arrington & Logan, 2005). Also, substantial interindividual differences emerge in terms of how much task selection is driven by exogenous factors (Arrington & Yates, 2009; Orr & Weissman, 2011), supporting a view of (partially) diverting processing streams. However, our results indicate that task-selection behavior incorporates the temporal predictabilities of tasks in forced-choice trials. This would also fit with the study by Mittelstädt et al. (2018) in which participants used the predictable waiting time for a repetition stimulus when deciding to switch or repeat tasks. A number of studies further substantiate the claim that bottom-up influences (that is, effects triggered by the task context and/or stimulus features) assert a huge effect on voluntary task choice (e.g., preparation time, stimulus repetitions, and task difficulty; Arrington & Logan, 2004; Mayr & Bell, 2006; Yeung, 2010). We suggest that the same may be true for the temporal predictability effects which we found here. In terms of the selection process, Herbort and Rosenbaum’s (2014) as well as Volberg and Thomaschkes (2017) studies suggest that action selection in this case, preparing the response rule and/or response hand associated with one task (Demanet & Liefooghe, 2014) is biased by the foreperiod–task contingencies, favoring compatible task choices.

This would also fit with our finding of a significant “short” task bias, that is, a bias toward the task associated with the short foreperiod. Even though this effect was considerably reduced when excluding participants with very strong task biases (that is with less variability in their task-choice behavior), the compatible choice bias still was larger after the short foreperiod. In our view, the emergence of time-based task-expectancy effects requires that participants first prepare for one task, and then switch to preparing for the other; it is highly likely that they fail more often to do so after the long foreperiod (see also De Jong, 2000). This idea is in accordance with the other findings of the literature. First, Pfeuffer et al. (2020) provide evidence that the frequency of anticipatory eye movements to the temporally predicted location (and task) is larger for a short compared to a long foreperiod. Second, Volberg and Thomaschke (2017) show that preparatory activity related to a certain temporally expected effector switches roughly at the expected end point of the short duration. Furthermore, Aufschnaiter, Kiesel, Dreisbach et al. (2018) found an RT benefit for the short compared to the long foreperiod in a temporally structured task environment. Relatedly, it is a well-known fact that participants tend to avoid switching tasks (Kessler et al., 2009), at least partially because of the effortful cognitive operations (e.g., reconfiguration of task sets) needed to implement those switches (Kool et al., 2010). The same may be true for temporally predictable tasks, where a switch in preparation after the short foreperiod has passed may equally be avoided.

Differential effects of foreperiod length can also be found in the variable foreperiod paradigm mentioned in the introduction (e.g., Steinborn et al., 2008), where sequential modulations can only be found with a current short foreperiod. Steinborn et al., (2009, 2010) used varying warning stimulus modalities (or features, Steinborn et al., 2010) to show that this sequential modulation was largely reduced when modality (or sufficiently distinct features within one modality) shifted, further providing evidence that short-foreperiod trials are influenced by preceding trials in a way that long-foreperiod trials are not: after-effects of the previous trial, e.g., its reinforced time point of peak readiness, thus seem to be limited to comparably short foreperiods. Other processes, such as conceptually driven, more intentional preparation processes, may prevail for longer foreperiods (Langner et al., 2018).

Similar effects can be seen in the task-switching domain, where interference from the previous task set is largest with no or very short preparation intervals (Meiran, 1996). Consequently, task-switching performance as well as voluntary switch rates increase with more time between trials (Arrington & Logan, 2004; Monsell & Mizon, 2006). In our paradigm, stronger compatibility effects were obtained for the short foreperiod and also the task associated with it. Thus, one could assume that while task selection of short-foreperiod trials depends on availability biases induced by the foreperiod-task contingency manipulation, any such biases are reduced the more time passes. Hence, the “short” task bias is a temporal bias in effect propagation of foreperiod compatibility: the compatibility bias may simply fade with time or become more noisy (as a sort of passive decay, cf. Meiran, 1996) or may sometimes be overruled by other factors impacting task choice (cf. Arrington & Logan, 2005; Langner et al., 2018).

The present results could also be interpreted in terms of an episodic-retrieval account (Hommel, 2004; Los et al., 2014; Mayr & Bell, 2006; Thomaschke & Dreisbach, 2015). According to this account, on each trial, a binding between the current foreperiod and the task is established that carries over to the next trial(s): a repetition of the current foreperiod automatically retrieves the task that was associated with it in the previous trial. Given that foreperiods and tasks in forced-choice trials were highly correlated, task-choice behavior in a following free-choice trial could simply reflect such an automatic retrieval of previous foreperiod-task bindings (for a similar argument, see Los et al., 2014) thus most likely result in a compatible choice. However, this account would predict more compatible choice repetitions than switches (cf., Mayr & Bell, 2006; Los et al., 2014), which we did not find in the current results. Nevertheless, episodic retrieval is suggested to be an aiding factor during learning of foreperiod–task associations (Thomaschke & Dreisbach, 2015) and could influence task choice in the current paradigm in addition to learned foreperiod–task associations. Future studies should directly test this account by contrasting trials where both foreperiod and task repeat or switch with trials were only one repeats.

### Temporal predictability effects on (voluntary) task performance

Importantly, we also found time-based task-expectancy effects on task performance. RTs and, somewhat attenuated, ERRs on free-choice trials mirrored the advantage of compatible task choice. Repetition and switch trials seemed to profit in a similar way by compatibility; or at least, with the current results, a clear dissociation is not possible (Exp. 1A: no effect for switch RTs; first two experiments: no effect for switch ERRs; Exp. 2: no effect for repetition ERRs). The similarity between task choice and task-performance results fits well with the preparation-switch account which we introduced earlier: time-based task expectancy acts on task preparation processes (as reflected in task-performance indices) that inform task selection (reflected in task-choice indices). However, the face validity of the performance-choice similarities has to be treated with caution—causal attributions cannot be made so far and further investigation is needed to corroborate this claim.

In a last 100% forced-choice test block in Exp. 1A and 1B, we checked whether effects of compatibility as established in the previous test phase would transfer to a block where foreperiod–task combinations were completely random. Here, we could show that indeed previously established foreperiod–task combinations were responded to faster and (only in the case of repetitions in Exp. 1A) more accurately. This reflects a replication and extension of the findings by Aufschnaiter, Kiesel, and Thomaschke 2018) who showed for forced-choice task switching that time-based task expectancy survives a change in absolute time environment. The current results extend these findings to a free-choice context.

### Implications for future research

Using different or a larger variety of foreperiods may be interesting with respect to the “short” task bias. Given that preparation in voluntary task switching as well as in foreperiod and interval timing studies is known to be successful only after some hundred milliseconds have passed, the question arises whether the “short” task bias would still be found if the short foreperiod was considerably shortened (e.g., to 100 ms). In this case, the default may rather be to start preparation only after this short foreperiod has passed given that only then task preparation (Kiesel et al., 2010) and accurate timing (Lewis & Miall, 2009) are possible. Furthermore, previous research on the variable foreperiod paradigm (Langner et al., 2018; Steinborn et al., 2009, 2010) has shown that the short-foreperiod “bias” in terms of susceptibility to sequential modulations can be largely increased when using more than two foreperiods as well as a greater range. In an experimental setup that was optimized for revealing differential effects of foreperiod length, Langner et al.’s (2018) Experiment 2 employed foreperiods of 800 ms, 1600 ms, and 2400 ms. Note that using more than two foreperiods also means that participants have to not only learn two foreperiod–task associations, but three. While increasing the overall task demand, this would also allow to investigate other task-switching phenomena in the context of temporal predictability, such as backward inhibition (Koch, Gade, Schuch, & Philipp, 2010).

This methodological approach may be informative in more than one respect: it may also allow to investigate whether the effect of temporal predictability on task-choice behavior is based on relative or absolute timing. Previous studies on time-based expectancy effects on performance seem to prompt the relative timing idea ((Aufschnaiter, Kiesel, & Thomaschke, 2018; Thomaschke, Kunchulia, & Dreisbach, 2015). That is, participants learn that one event appears after the interval that is relatively shorter/longer than another interval, rather than learning the correlation between the exact time period and the event. If we think of the example in the beginning of waiting for an unpunctual friend, relative timing information seems sufficient for changing the anticipated course of action: the more time passes, the likelier it is that the person will not show up and I will have to act accordingly. (Aufschnaiter, Kiesel, & Thomaschke, 2018 make the legitimate claim that finding time-based task expectancy to only involve relative timing information may be due to the experimental design involving only two foreperiods. Given that many real-life scenarios require absolute timing (e.g., a pilot operating in a cockpit), it may be a fruitful endeavor to see whether time-based task-expectancy effects, particularly on task-choice behavior, can be shifted toward absolute timing.

## Conclusion

The present experiments show, for the first time, that time-based task expectancies influence free choice. The current research extends findings which showed that contingencies between onset latency and tasks can be learned (Aufschnaiter, Kiesel, Dreisbach et al., 2018; Aufschnaiter, Kiesel, & Thomaschke, 2018) and will influence performance, by extending these to a free-choice environment: both for choice and performance parameters, an advantage for predicted foreperiod–task combinations was found. The fact that these effects were more pronounced for the short foreperiod suggests that participants started out each trial with preparing for the task associated with the short foreperiod and, if it passed without a target appearing, were not always able to switch preparation to the other task. On a more general level, the current research adds to existing findings demonstrating contextual influences on voluntary task switching (Mayr & Bell, 2006; Mittelstädt et al., 2018; Yeung, 2010).

## Footnotes

1 It has been shown that differences in task difficulty induce a task bias toward the easier task under unrestricted free choice (Jurczyk et al., 2018), and that the three-digit number and the alphabet letter task are comparable in task difficulty without inducing a task bias Fröber and Dreisbach (2017).

2 Boxplots were created using the SPSS built-in tool with mean RT and ERRs collapsed across all design cells. By default, values deviating more than three interquartile ranges from Q3 or Q1 are marked as outliers. Exclusion of these participants had no impact on the statistical pattern of results obtained.

3 In post hoc analysis, we excluded participants who displayed extreme task biases. A cut-off value of 95% was chosen based on the distribution of task choice rates, as displayed via histograms: while the majority of data points could best be described by a widespread but rather smooth bell-shaped distribution around the 50% value with gradually less occurrences the more extreme the task choice, seven participants were clotted at the end points at over 95% task bias to either task. Note that five of these participants had a task bias toward the “short” task irrespective of whether it was a number or letter task. When excluding those participants, the “short” task bias was no longer significant (*M* = 54.2, *SD* = 19.8), *t*(23) = 1.04, *p* = .311, *d* = 0.21, while the compatible choice rate still was (*M* = 56.3, SD = 8.2), *t*(23) = 3.78, *p* < .001, *d* = 0.77.

4 Here and hereafter, we will use the term “short” task bias whenever participants display a bias—that is, a choice rate of > 50%—toward the task that is associated with the short foreperiod on forced-choice trials. This might either be the number or letter task depending on the particular participant (as foreperiod-task assignments were counterbalanced). In particular, we want to emphasize that it does not necessarily refer to the task for which participants show shorter RTs.

5 Of 32 participants, ten reported that they were aware of the foreperiod-task contingencies. Excluding these participants from the task-choice analyses did not alter the statistical pattern of results obtained—in particular, the compatible choice bias was still significant (*M* = 55.6%), *t*(21) = 3.58, *p* = .002, *d* = 0.76. Thus, the additional analyses excluding those participants suggest that awareness was not vital for the effects obtained.

6 Note that similar to Experiment 1A, all of these participants had a task bias toward the “short” task irrespective of whether it was a number or letter task.

7 Six of all 32 participants indicated that they grew aware of the foreperiod manipulation. The statistical result pattern was basically identical when excluding these participants.
